# Selected Physiological Effects of a *Garcinia Gummi-Gutta* Extract in Rats Fed with Different Hypercaloric Diets

**DOI:** 10.3390/nu10050565

**Published:** 2018-05-04

**Authors:** Carolina Guillén-Enríquez, Veronica Lopez-Teros, Ubicelio Martín-Orozco, José A. López-Díaz, Julio del Hierro-Ochoa, Arnulfo Ramos-Jiménez, Humberto Astiazarán-García, Nina del Rocío Martínez-Ruiz, Abraham Wall-Medrano

**Affiliations:** 1 Instituto de Ciencias Biomédicas, Universidad Autónoma de Ciudad Juárez, Anillo Envolvente del PRONAF y Estocolmo s/n, Ciudad Juárez 32310, Chihuahua, Mexico; carolina.guillen@uacj.mx (C.G.-E.); umartin@uacj.mx (U.M.-O.); joslopez@uacj.mx (J.A.L.-D.); jdelhier@uacj.mx (J.d.H.-O.); aramos@uacj.mx (A.R.-J.); 2 División de Ciencias Biológicas y de la Salud, Universidad de Sonora, Hermosillo 83000, Sonora, Mexico; veronica.lopez@unison.mx; 3 Centro de Investigación en Alimentación y Desarrollo, AC (Unidad Hermosillo), Coordinación de Nutrición, Carretera a la Victoria km. 0.6, AP 1735, Hermosillo 83000, Sonora, Mexico; hastiazaran@ciad.mx

**Keywords:** Garcinia cambogia, Western diet, high-fat diets, hydroxicitric acid, DXA, obesity

## Abstract

*Garcinia gummi-gutta* (GGG) rind extract is effective for reducing appetite, body weight and adiposity of obese rodents fed high-fat (HF), high-sugar (HS) or high fat/sugar (HFS)-based diets, but these effects have not been simultaneously evaluated. Thirty obese (~425 g) male Wistar rats were fed for eleven weeks with six hypercaloric diets (4.1 kcal/g; five rats/diet) non-supplemented (HF, HS, HFS), or supplemented (HF+, HS+, HFS+) with GGG extract (5.9%), while rats from the control group (375 g) were fed a normocaloric diet (3.5 kcal/g). Body weight, dietary intake, body fat distribution, and histological and biochemical parameters were recorded. Compared to control rats, non-supplemented and supplemented groups consumed significantly less food (14.3% and 24.6% (−4.3 g/day), respectively) (*p* < 0.05). Weight loss was greater in the HF+ group (35–52 g), which consumed 1.9 times less food than the HS+ or HFS+ fed groups. The HF and HFS groups showed 40% less plasma triacylglycerides and lower glucose levels compared to the HF+. GGG-supplemented diets were associated with lower ketonuria. The HF+ diet was associated with the best anti-adiposity effect (as measured with the dual X-ray absorptiometry (DXA) and Soxhlet methods). The severity of hepatocyte lipidosis was HF > control > HF+, and no signs of toxicity in the testes were observed. The results indicate that GGG is more effective when co-administered with HF diets in obese rats.

## 1. Introduction

Overweight/obesity (BMI ≥ 25 kg/m^2^) has become the most important public health burden worldwide. In 2017, the Organization for Economic Cooperation and Development stated that one out of five adults (3.7% (Japan)–38.2% (USA)) around the globe suffer from obesity and its complications, a phenomenon that will steadily increase until 2030 [1]. Excess body weight is a prodromal condition for both morbidity and mortality which is associated with different non-communicable chronic diseases such as type 2 diabetes, ischemic heart disease, and cancer, all of which reduce the quality of life among patients [2]. From a physiological point of view, weight gain results from an excessive accumulation of fat (e.g., abdominal area) as a consequence from an imbalanced energy intake/expenditure. Many pharmacological (e.g., Orlistat^®^), dietary (e.g., low-fat/low-carbohydrate dietary regimes) and physical activity-based interventions aimed at reducing body fat stores have been shown to be effective under strict clinical supervision [3,4,5]. However, most of those strategies rely on people’s behavioral changes towards a healthier lifestyle, compromising adherence if behavioral changes are not achieved [6]; moreover, pharmacological therapies are sometimes inefficient, expensive and may cause negative side effects [3]. Consequently, overweight/obese subjects are continuously seeking practical alternatives to improve their body weight, turning to over-the-counter (OTC) nutritional supplements, which are widely accepted by these users, leading to a specific market segment of nearly 630 million USD in direct sales and an infrastructure (supermarkets and drugstores) of nearly two billion USD in 2015 with an expected growth of 5% (4.8 million in revenues) by 2020 [7].

*Garcinia gummi-gutta* (GGG; *G. cambogia*) rind extract is a nutraceutical preparation aimed at reducing appetite, fat stores and body weight [8,9,10]. These effects are attributed to (−)-hydroxycitric acid (HCA), a major organic acid present in GGG’s rind (covering shell; 50–60%, *w/w*), which competitively inhibits cytosolic ATP-citrate lyase, which, in turn, catalyzes the synthesis of acetyl-CoA and oxaloacetate using citrate, CoA, ATP (substrates), and Mg^2+^ (cofactor). This enzymatic reaction precedes the hepatic biosynthesis pathway of fatty acids, cholesterol and triacylglycerides; additionally, HCA can lower the plasmatic lipid concentration through the inhibition of cholesteryl ester transfer protein [11].

Short-term weight-loss, anti-liver steatosis and lipid-lowering effects of GGG rind extracts have been reported in rodents and humans, showing mild or no side effects when a convenient pharmacological dose was used [8,9,10]. Particularly, the metabolic outcomes described above have been been observed for rodents fed different concentrations of high fat (HF), or high sugar (HS)-based diets, either alone or combined (HFS; “Western”) and using different experimental designs [12,13,14,15,16,17,18]. Thus, an objective comparison of GGG’s HCA benefits on the metabolic derangements associated with primary disturbances in adipose tissue, promoted by these diets in one single experiment has, to our knowledge, not been addressed. Therefore, the aim of this study was to assess the effect of a standard supplementation (5.9 g/100 g) of a GGG rind extract (60% HCA) on the metabolic effects of three hypercaloric (4.1 kcal/g) diets (HF+, HS+, HFS+), as compared to their non-supplemented counterparts (HF, HS, HFS), in diet induced-obese rats.

## 2. Materials and Methods

### 2.1. Experimental Design

Male Wistar rats (~250 g; *n* = 35) were provided by the animal care facility of the Universidad Autónoma de Ciudad Juárez. The experimental design is summarized in Figure S1 (Supplementary Material). Briefly, the experimental phases were (A) Acclimatization, in whic rats were housed individually in polycarbonate cages under controlled environmental conditions (22 ± 2 °C, relative humidity 45–60%, 12 h light to dark cycles) and fed with a standard rodent diet and water ad libitum for three days; (B) Obesity induction, in which animals were assigned to a normocaloric (“control”, 3.5 kcal/g; *n* = 5) growth diet AIN-93M [19] or a hypercaloric (5.2 kcal/g; 61.2% energy from fat: soybean oil (10%) and lard (90%); *n* = 30) diet with 1% cholesterol (mimicking a D12492 diet; Research Diets, Inc., New Brunswick, NJ, USA) which was maintained for five more weeks in order to develop obesity + mild dyslipidemia in the hypercaloric group [20]; and (C) Bioassay, in which obese rats were randomly assigned to one of six hypercaloric diets (4.1 kcal/g), either alone (groups: HF, HS, HFS) or supplemented (groups: HF+, HS+, HFS+) with a GGG rind extract. The amount of GGG rind extract added (5.9 g/100 g diet, 60% HCA) was selected to maximize the average intake of HCA [12,13,14,15,16,17,18] in order to observe clinical benefits without causing adverse effects [8,9,10]. The groups maintained the assigned diet for eleven more weeks, while rats fed the “control” diet kept the same normocaloric diet without GGG supplementation. Details on the final composition (g/100 g) and the caloric macronutrient distribution (%) of total energy intake are described in Table 1 and Figure S2 (Supplementary Material), respectively.

Animals and residual diets were weighed daily using an electronic scale (Tor Rey^®^, EQ-4HP, Torrey, Mexico City, Mexico) to the nearest ±0.1 g, and food intake was corrected for spillage. At the end of the study and after an overnight fast, animals were sacrificed under anesthesia (Zelazol^®^, 30 mg/kg BW (body weight); Zoetis, Mexico City, Mexico) by cervical dislocation. All experimental procedures were approved by the Institutional Committee of Ethics and Bioethics of the Universidad Autónoma de Ciudad Juárez (CBE.ICB/039.03-15), following the national legislation on the use of laboratory animals (NOM-062-ZOO-1999) and the National Institutes of Health (NIH) Guide for Care and Use of Laboratory Animals.

### 2.2. Biological Samples

Blood samples were collected in EDTA-coated tubes, and plasma was recovered by centrifugation (2000× *g*, 10 min, 4 °C) and stored at −80 °C until its use. Organs (viscera, liver and testes) were carefully removed under anesthesia, rinsed with sterile PBS (phosphate-buffered saline) (0.85%), blotted on filter paper to remove excess water, and weight was recorded. Small sections of the organs were either fixed in formalin/phosphate buffered saline (10-fold volume) for at least 48 h and/or stored in sealed plastic bags at −20 °C until their use. Individual urine samples were collected every two weeks throughout the study and freeze-dried until analysis.

### 2.3. Biochemical Analyses

Plasma concentrations of glucose (fasting glucose (FG); Spinreact, Girona, Spain), triacylglycerides and total cholesterol (TAG/TC; LiquiColor^®^, Stanbio Laboratory, Boerne, TX, USA), and high density lipoprotein cholesterol (HDL-C, BioSystems^®^, Biosystems S.A., Barcelona, Spain) were measured using standard enzymatic-colorimetric methods, following the manufacturers’ instructions. Total lipids from dried viscera (105 °C, 48 h), hepatic, and fecal samples were extracted and quantified using the Soxhlet method (Soxtec^TM^, mod. 2043 FOSS, Eden Prairie, Minneapolis, MN, USA) and expressed as g/100 g of sample. Urine samples were collected to quantify ketone bodies (KB) using reactive strips (Combur 10Test^®^ UX Cobas^®^, Roche Diagnostics International Ltd., Tucson, AZ, USA) and reflectance photometry (Miditron^®^, mod. Junior II, Roche Diagnostics, Indianapolis, IN, USA). All assays were performed in quadruplicate.

### 2.4. Dual X-ray Absorptiometry (DXA)

Total and regional (abdominal, thigh, visceral areas) body fat depots were measured using a Hologic QDR-2000W densitometer (Hologic, Inc., Bedford, MA, USA) a week before sacrifice. Each rat was anesthetized, as described above, and positioned for scanning conditions, which were performed as follows: dual energy 140/100 kVp, 2.5 mA average, 122 s and 60 Hz [21]. The anatomic regions examined were the abdomin (R1; rectus), thigh (R2, hip and hind leg), visceral and total body (∑R1+R2+visceral). Fat mass is expressed as percentage of body weight.

### 2.5. Histology

Liver and testes were fixed in 10% neutral buffered formalin, embedded in paraffin, and cut into 3- to 5-μm sections. Each set of tissues was stained with hematoxylin and eosin [22]. Hepatic samples were graded for non-alcoholic steatohepatitis (NASH), as suggested by Brunt et al. [23], to rate the severity and localization of the following histological features: steatosis, ballooning, fibrosis, and intra-acinar/portal inflammation [23]. The rind extract toxicity of GGG was considered positive when spermatids, hydropic degeneration and edema were found in the testes. Micrographs were taken at 400× magnification, with a photomicroscope (Axio Lab. A1, Axiocam 105, Zeiss, Jena, Germany) and processed with ZEN 2.3 blue edition software (Zeiss, Jena, Germany).

### 2.6. Statistical Analysis

Data is presented as means ± SDs (standard deviation). Normally distributed data was analyzed using Levene’s test and one-way analysis of variance (ANOVA) using Fisher’s multiple comparisons (LSD); when Levene’s test was statistically significant, unequal variance t-Student tests were used. To test the effects of food intake between groups, analysis of covariance (ANCOVA) (type III sum of squares) using Fisher’s LSD was also used. Non-parametric variables were analyzed using the Mann–Whitney U and Kruskall–Wallis tests. All statistical analyses were conducted using XLSTAT (version 2015.6.01; Addinsoft, Paris, France). Statistical significance was defined when *p* < 0.05.

## 3. Results

### 3.1. Bioassay Performance

Table 2 shows the changes in body weight and dietary intake of the rats from the different intervention groups. Initial body weights were higher for the rats from the non-supplemented (47–59 g) and supplemented groups (39–58 g; except HFS+), compared to the control rats, placing them above the 97th body weight percentile [20].

On average, obese rats fed with non-supplemented diets (HF, HS and HFS) had lower food intakes (1.8–2.9 g/day) than those observed for control rats, which led to less weight gain in the 11th week; this difference was statistically significant only for HS and HFS-fed rats vs. the control group (−30.6 g). On the other hand, rats fed with GGG-supplemented diets had lower food intakes compared to control rats (3.6–5.5 g/day; *p* < 0.05) or their non-supplemented counterparts (1.9–2.8 g/day), except for HFS+/HFS-pair fed groups. The appetite-reducing effect was observed as follows: HF+ (19%; *p* < 0.01) > HS+ (11.5%; *p* = 0.03) > HFS+ (6.5%; *p* > 0.05). It is noteworthy that HF+ fed rats consumed ~1.9 g/day less food and 0.1 g/day less GGG rind extract than HS+ and HFS+ fed groups, a result associated with a greater weight loss in HF+ rats (−47 ± 24 g) compared to the final body weights of the control rats.

### 3.2. Biochemistry

Plasma and urine biochemical analyses are shown in Table 3, where the results for TAG were lower for HF (28.8%; −60 mg/dL) and HFS (40.4%; −100 mg/dL) groups compared to HS and control groups, respectively. The control group had a significantly higher concentration of HDL-C (43.0 ± 7.3 mg/dL), but there was no statistical difference for total cholesterol (TC) and HDL-C levels (~120 and 34.5 mg/dL) among non-supplemented groups. Additionally, ketone body excretion by rats in the non-supplemented groups was significantly higher than that in the control group (3.5 ± 1.8 mg/dL).

On the other hand, TAG concentrations in the GGG-supplemented HS+ group were lower than those observed in the HS group. Glucose concentrations were significantly lower for HF+ fed rats, as compared to those in the HF and HS+ groups. However, TC and HDL-C did not differ among GGG rind extract-supplemented groups (~118 and 29.5 mg/dL). The urinary excretion of ketone bodies was significantly lower in the HF+ and HS+ groups compared to their non-supplemented counterparts (HF and HS) by 70% and 49%, respectively.

### 3.3. Body Fat Distribution

There was no significant difference in fat composition (as % body weight) among obese rats for the total, abdominal and thigh areas, except for visceral (+3.4 g) and hepatic (+6.8 g) fat depots (*p* < 0.05), when compared to the control group (Table 4). It is noteworthy that, although the pattern of fat distribution was homogenous, the control rats’ body weights were less than the non-supplemented rats’ body weights, and therefore, total fat mass was much higher, regardless of the area evaluated. Additionally, the fecal excretion of fat from the non-supplemented groups was twice that observed in the lean (control) rats (1.0 ± 0.4 g/100 g dry weight), and hepatic fat deposits were once (HS) or twice (HF, HFS) the amount found in the control group. On the other hand, body fat distribution was similar among GGG rind extract-supplement groups. 

In the comparison between GGG rind extract-supplemented vs. non-supplemented paired-fed groups, HF+ showed the highest anti-adiposity effects (8–29% reduction; g/100 g BW) in the following order: abdominal (−20.3 g) > total (−15.1 g) > thigh (−12.0 g) > visceral (−12.4 g). Also, the fat content in liver (as assayed by the Soxhlet method) was lower in the HF+ (43.9%), HFS+ (24.8%) and HS+ (11.4%) groups compared to their respective non-supplemented groups. Even though the GGG rind extract-supplemented groups had higher fecal fat excretion (range: 120% (HS+)–140% (HF+)) compared to the control group, no difference was observed with their non-supplemented counterparts (Table 4).

### 3.4. Histological Examination

**Figure 1:**
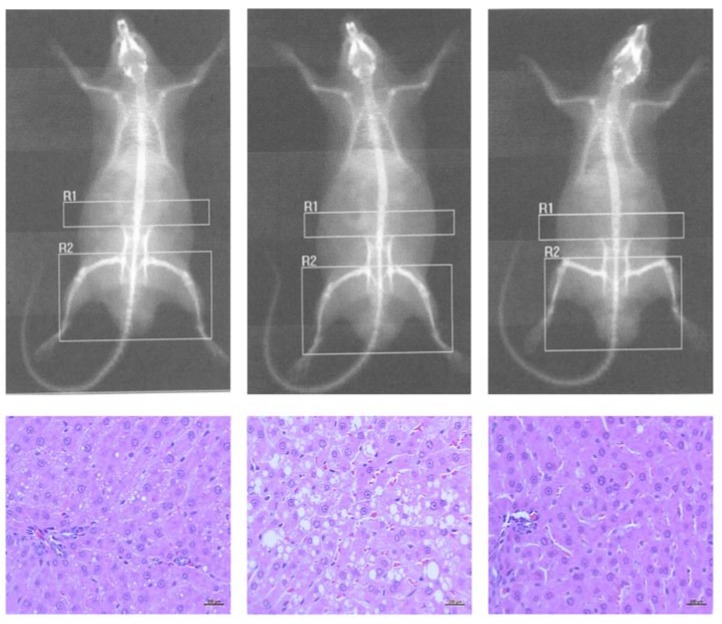
Imaging (DXA) and histological (H&E) findings. Control (left) and non-supplemented high-fat (HF; center) group and the group supplemented with GGG rind extract (HF+; right). Liver micrographs were captured at 400×. DXA conditions: dual energy 140/100 kVp, 2.5 mA average, 122 s, 60 Hz.

Consistent with the DXA findings, liver samples from the HS and HFS-fed groups showed a different pattern of hepatic lipidosis (macro-to-microvesicular fat droplets) than those from the HF-fed rats (Figure 1, bottom-center), although this qualitative observation was not statistically different between groups. Also, hepatic inflammation and ballooning were observed in some cases (data not shown), but other NASH- or fibrosis-related features were not common [20]. Consistent with the anti-adiposity effect observed in the HF+ group (8–29%; as evaluated by DXA), the severity of hepatocyte lipidosis was HF > control > HF+ (Figure 1). Additionally, the control group (left-down) showed expanded hepatocytes in the periportal zone with few lipid droplets (mild lipidosis), and the non-supplemented HF rats (center-down) showed expanded mid-zonal lesions with macro and micro-vesicular droplets (severe hepatic lipidosis; fatty liver); furthermore, lesions characteristic of fatty liver were attenuated in the GGG-supplemented HF+ group (right-down to a higher extent than in the control group. Lastly, testicular tissues from GGG rind extract-fed groups did not show any lesions associated with the acute/chronic toxicity of HCA.

## 4. Discussion

### 4.1. Physiological Effects of Hypercaloric Diets

Many epidemiological studies show the strong relationship between certain “unhealthy” dietary patterns and weight gain and body adiposity [24,25]. A close examination of people’s eating behaviors (e.g., meal sizes, eating occasions and rate) and the presence of eating disorders (e.g., binge eating) is essential to understand the metabolomic impact of specific nutrients on the development of obesity. For example, the so-called “Western diet” (HFS) is characterized by a high intake of processed foods, oils and fats, and sugar-sweetened beverages, and a reduced intake of fruits, vegetables and grains, resulting in a high glycemic load, redox imbalance and low dietary intake of micronutrients that is often found in individuals with morbid obesity and/or metabolic syndrome [26].

Even before food touches the mouth, physiological signals are generated by the sight and smell of food (e.g., salivation), in both humans and rodents, as a first step to prepare the body for the uptake (digestion/absorption) of nutrients and their further deposition and excretion. In that sense, animal models (e.g., rats) fed HF, HS and HFS-based diets have been used for decades to mimic human obesity, dyslipidemia, insulin resistance and metabolic syndrome [27,28,29]. However, the physiological response to each of them is quite different. For instance, male C57BL/6 mice fed with diets differing in their fat-to-carbohydrate ratio (standard lab chow (10–14% calories from fat), HF (50% calories from fat), Western (42% calories from fat + 0.2% cholesterol), high-fructose (60%, *w/w*)) have variable epigenetic responses in multiple genes for epithelial transporters, phase I/II enzymes and transcription factors that regulate lipid and carbohydrate metabolism [30,31,32], partially explaining why the obesity phenotype and cardiometabolic risk of C57BL/6 mice differ according to the type of diet [33].

In this study, a clear appetite-reducing effect was observed with HF and HFS diets compared to lean (control) and HS-fed rats. It has been reported that diets with a metabolizable energy ranging from 3.7 to 5.4 kcal/g are highly palatable and induce obesity in rats [29]; however, food intake decreases as soon as body weight increases [17,34], particularly when diets are formulated as ≥5.2 kcal/g, causing a 22% reduction in food intake [35]. Leptin concentration may play a role in the reduction of food intake and energy expenditure in obese rats fed HF diets by means of a hypothalamic glucose-sensing mechanism [36], given that the release of this hormone from adipose tissue has been shown to positively correlate with total body fat in male Wistar rats (*p* < 0.001) [37]. Despite the appetite-reducing effect and the lower absolute weight gain observed in non-supplemented obese rats (HF, HS, HFS groups), by the conclusion of the study, they weighed more, had higher visceral (mainly hepatic) fat deposition and excreted twice as much urinary ketones and fecal fat than lean (control) rats. These findings support the idea of a lower fat mobilization from adipose/hepatic tissues, concurrent with a higher rate of beta-oxidation [38]. A study carried by Dominguez-Avila et al. [37], fed young male Wistar rats (148 g BW) for 9 weeks with a control diet (3.6 kcal/g; 25.3% from fat) and a HF-diet (4.5 kcal/g; 54.8% from fat), and results showed a lower food intake, higher plasma leptin levels, higher body and hepatic fat content with mild or no lipidemic modifications (TC, HDL-C, TAG, low-density lipoprotein cholesterol (LDL-C)) in the HF group, similar to our findings for the HF and control groups. Regarding the higher fecal fat excretion, several protein-mediated (e.g., CD36 and FATP4) and simple-passive diffusion mechanisms used for the intestinal absorption of lipids have been reported, both of which function in a dose-dependent manner in a way that the intake of HF or HFS diets could have induced a “saturation” phenomenon [38].

Thus, the metabolic effects described above were particularly strong in the HF group, who developed fatty liver and whose circulating TAG were on the lower end. It is widely accepted that the liver plays a major role in regulating the body’s lipid homeostasis, since minor changes in hepatic fatty acid and TAG metabolism have diverse effects on the hepatic fate of other nutrients (e.g., sugars), in generating hypermetabolic states of peripheral organs, and in the development of many metabolic diseases [39]. HF diets also promote fatty liver due to an imbalance of TAG synthesis, breakdown (oxidation), trafficking, transport, lipoprotein sythesis, and hepatic REDOX homeostasis [37,38]. It is noteworthy that the extent of liver damage (fatty liver, NASH or non-alcoholic fatty liver disease) in rodents depends on factors, such as the macronutrient dietary distribution and feeding protocol (acute, sub-chronic, chronic), that said, the feeding protocol and the different diets used in this study do not seem to have induced metabolic dearrangements other than macro-/micro-vesicular fat deposition in a diet-dependent manner.

### 4.2. Physiological Effects of GGG Rind Extract Co-Administered with Hypercaloric Diets

Several countries are adopting initiatives to prevent obesity and to improve the quality of life of people who suffer from its complications, either with primary (lifestyle modifications) and/or secondary (prescribed drugs) interventions [2]. Although lifestyle modifications (i.e., healthy eating habits and physical activity) are the preferred options for reducing/controlling body weight, obese subjects often fail to complete such interventions [6]. Therefore, health professionals often advise patients to consume OTC supplements as an efficient way to manage their weight and to reduce the risk of non-communicable diseases and disabilities. Within the functional food and nutraceutical market, weight-loss supplements are the fastest growing segment, with an expected compound annual growth rate of 5.6% in the following five years [7]; these supplements are classified into four categories depending on their mechanism of action: (i) blockers of lipid and carbohydrate absorption; (ii) thermogenic products; (iii) products that change body composition; and (iv) appetite suppressors or satiety inducers [40]. According to this classification, GGG rind extract may exert all mechanisms, except for inhibiting digestive enzymes [41].

The combination of GGG rind extract with HF+ and HS+ diets reduced food intake beyond the reduction observed in the non-supplemented hypercaloric diets (HF and HS). We would like to highlight that our results showed that HF+ fed rats consumed less GGG rind extract (0.1 g/day) than the HS+ and HFS+ fed groups, which would translate as a limited effect from HCA, but this did not occur. The effect of HCA on reducing food intake (rate: 0.9 g·day^−1^/g of HCA; *R*^2^ = 0.95) and inducing weight loss (rate: −6.3 g BW/g of HCA; *R*^2^ = 0.93) in rats has been known since the early 1970s, when Sullivan et al. [42] reported dose- and duration-dependent effects of HCA (as trisodium salt) when administered intragastrically to female CD rats (195 g) fed a HS diet (70% glucose); the authors attributed these benefits to HCA’s interference on “channeling glucose (and intermediary metabolites) to fatty acid and cholesterol biosynthesis”, a mechanism by which HCA competitively inhibits ATP-citrate lyase, regardless of the type of diet provided [8,9,10]. Brandt et al. [17] found similar results when evaluating the acute supplementation (4 weeks) ad libitum of a high-sugar (50% fructose) diet with (HS+) or without (HS) HCA (1.8%, *w/w*) in rats. Because in our study appetite suppression and weight-loss were diet-dependent (HF+ > HS+ > HFS+), we conclude that the macronutrient composition of experimental diets plays a major role when evaluating the effectiveness of GGG rind extract for weight-loss. Aside from HCA’s well-known effect on the rate of lipogenesis upon fat/carbohydrate bioavailability, novel metabolic actions like the cholesteryl ester transfer protein (CETP) inhibitor [11] may also explain this phenomenon [43].

Available literature shows that HCA may be toxic, but a review by Chuah et al. [44] reported a “no observed adverse effect level (NOAEL)” at up to 2.8 g/day, while Ranjith et al. reported no drug-related mortality and toxicity (biochemical and histopathological analysis) showing a LD_50_ of pure HCA of 5000 mg/kg body weight in Wistar rats [45]. This evidence leads us to conclude that HCA is safe for use, which supports the results observed in this study by the histological evaluation of testicular tissues from HF+, HS+ and HFS+ groups.

Moreover, the differential bioavailability ratios of dietary fat/carbohydrates when co-administered with the GGG rind extract caused a mild lowering effect on glucose and TAG concentration in HF+ and HS+ fed rats, respectively, when compared to their non-supplemented counterparts (HF, HS). The lipid-lowering effects of GGG rind extracts have been extensively documented in both rodents and humans, with mild or no side-effects when used at a convenient pharmacological dose [8,9,10]. Unfortunately, reports on the hypoglycemic mechanisms exerted by GGG on HCA are scarce. Brandt et al. [17] found that HCA did not ameliorate hypertriglyceridemia, plasma insulin or liver glycogen levels when co-administered with a HS diet (50% fructose *w/w*); however, in chicken embryos, HCA significantly increased plasma glucose but also hepatic glycogen [46], implicating some sort of efficient anabolic utilization of glucose that should be further investigated. Also, compared to non-supplemented diets, urinary ketones were substantially reduced with GGG rind extract-supplemented diets, although the fecal fat excretion did not change. From a metabolic point of view, ketonuria results from higher biosynthesis of ketone bodies (acetoacetate and beta-hydroxybutyric acid) under specific metabolic conditions such as starvation and active lipolysis, such as that achieved with the intake of HCA. On the other hand, the lack of effect of GGG rind extract on fecal fat excretion is likely due to its low inhibitory capacity on pancreatic lipase [41].

The best anti-adiposity effect (evaluated by DXA) was seen in the HF+ group (12–20%) and the severity of hepatocyte lipidosis was HF > control > HF+. Oral administration of HCA significantly reduces in vivo lipogenic rates in a dose-dependent-manner in the liver, adipose tissue and small intestine of rats, a process involving ATP-citrate lyase inhibition [43], while in chicken embryos, HCA inhibits TAG hepatic synthesis by well-orchestrated events that include accelerated lipolysis by enhancing lipoprotein-lipase, hepatic-lipase, and transactivation of the adiponectin signaling pathway [46]. Also, in broiler chickens, HCA reduces abdominal fat deposition by regulation at the transcriptional level, not only ATP-citrate lyase but also fatty acid synthase, PPARα, and SREBP-1c [47]; even when those mechanisms are plausible, the epigenetic effects in normal and obese rats deserve further investigation. Our results showed a small difference between body fat depots in HS+ and HFS+ fed groups vs. their non-supplemented counterparts, but a study carried by Brandt et al. [17], showed that when testing the effects of HS (50% fructose) with (HS+) or without (HS) HCA during a 4 week period, lower visceral fat accumulation in HS+ group was observed. However, the hepatic fat content in the HS+ rats was higher that in non-supplemented HS rats; the authors also found a mild reduction of plasma insulin levels (day 10), which increased rapidly until the end of the study; thus, hormonal adaptations may be responsible for the lack of effect observed in our HS+ and HFS+ fed rats.

Finally, the authors recognize the study’s limitations which included the lack of: (A) a more comprehensive evaluation on the effect of the intervention at tissular level using molecular and genetic biomarkers (e.g., circulant adipokines, liver gene expression/protein levels of pro-adipogenic mediators) and (B) pharmakokinetic assays to evaluate bioavailable phytochemicals (and secondary metabolites) after GGG rind intake (acute/chronic exposure). Including those biometric studies would allow a more in-depth analysis and an insightful study; thus, a plausible physiological mechanism associated with the effect of the GGG rind extract described here cannot be drafted from current data. Nevertheless, studies reporting similar results have elucidated certain metabolic activation/inhibition pathways related to GGG rind’s HCA content, all of which were discussed above. In any case, our study could be used as the basis to perform novel studies with a stronger metabolomic/chemometric design.

## 5. Conclusions

New scientific evidence regarding the differential effects of GGG rind extract (60% HCA) when co-administered with hypercaloric diets (4.1 kcal/g) to obese rats, were reported in this manuscript. Results indicate that the GGG rind extract used (5.9%, 60% HCA) is more effective for losing weight and body fat when co-administered with HF diets than HS+ or HFS diets. Additionally, hypercaloric diets with different macronutrient profiles caused a different metabolic impact in rodents [48,49,50], and the GGG-rind extract supplement had a differential effect when co-administered. Moreover, the observed benefits were achieved with no side effects or toxicity at a dose of 0.71–0.81 g/day of GGG rind extract. Translating research findings from murine bioassays into human trajectories of disease and therapeutics has been one of the major challenges of translational medicine, but, from an ethical point of view, pharmacological testing in murine models precedes clinical trials in humans, and for GGG rind extracts, the evaluation of its pharmacological action, nutraceutical effects, and toxicity in murine models has been useful for this effect [12,13,14,15,16,17,18]. Nonetheless, all findings reported in this study should be further assessed in an intervention trial in humans.

## Figures and Tables

**Table 1:** Experimental diets (g/100 g).

Ingredient	Control	Non-Supplemented ^5^			Supplemented ^5^ (+)		
		HF	HS	HFS	HF+	HS+	HFS+
GGG rind extract ^1^	-	-	-	-	5.9	5.9	5.9
Soybean oil ^1^	4.0	20.7	15.0	20.7	20.7	15.0	20.7
Raw sugar ^1^	9.0	9.0	51.9	30.4	9.0	51.9	30.4
Corn starch ^1^	56.8	35.0	4.9	13.8	35.0	4.9	13.8
Casein ^2,3^	12.1	12.1	12.1	12.1	12.1	12.1	12.1
Cellulose ^2^	5.0	5.0	5.0	5.0	5.0	5.0	5.0
AIN93G-mineral mix ^2^	1.0	1.0	1.0	1.0	1.0	1.0	1.0
AIN93-vitamin mix ^2^	3.5	3.5	3.5	3.5	3.5	3.5	3.5
Choline chloride ^4^	0.2	0.2	0.2	0.2	0.2	0.2	0.2

^1^ Food-grade (several trademarks): *Garcinia gummi-gutta* (GGG) rind extract, 60% (−)-hidroxicitric acid (HCA) (Xi’an Nate Biological Technology Co., Ltd., Xi’an, Shaanxi, China); soybean oil (Nutrioli^®^, Regasa, Monterrey, Mexico), raw sugar (sucrose + maltodextrins); ^2^ Food-grade ingredients from Bioserv, Inc. (Frenchtown, NJ, USA), ^3^ ANRC grade, vitamin free, >0.98 g total sulfur amino acids (TSA)/100 g diet; ^4^ 99% pure (74.6% choline); ^5^ All but control diet (3.5 kcal/g) were hypercaloric (4.1 kcal/g) with added water (6.5–13.5 g/100 g) enough to reach a 100% formulation: High fat (HF), high sugar (HS), western (high fat/sugar; HFS) with or without GGG rind extract (HF+, HS+, HFS+). Caloric macronutrient distribution is shown in Figure S1 (Supplementary Material).

**Table 2:** Bioassay performance of rats fed with experimental diets (11 weeks).

Diet	Body Weight (g)			Dietary Intake (g/day)	
	Initial	Final	Δ Change	Food	GGG
Control ^1^	374.6 ± 22.3 ^b^	455.8 ± 23.6 ^ab^	81.2 ± 21.6 ^a^	17.5 ± 1.2 ^a^	-
HF ^2^	425.6 ± 13.7 ^a^	480.4 ± 30.8 ^a^	54.8 ± 18.3 ^ab^	14.8 ± 0.9 ^bc^	-
HS ^2^	433.4 ± 33.4 ^a^	483.4 ± 45.5 ^a^	49.9 ±27.6 ^b^	15.7 ± 2.3 ^b^	-
HFS ^2^	421.7 ± 32.1 ^a^	473.1 ± 35.9 ^a^	51.4 ± 10.3 ^b^	14.6 ± 1.0 ^bc^	-
HF+ ^3^	423.3 ± 37.6 ^a^	376.3 ± 45.6 ^c^	−47.0 ± 23.6 ^d^	12.0 ± 1.2 ^d^	0.71 ± 0.07 ^b^
HS+ ^3^	432.9 ± 30.2 ^a^	416.6 ± 30.2 ^bc^	−16.3 ± 21.3 ^c^	13.8 ± 0.9 ^c^	0.81 ± 0.05 ^a^
HFS+ ^3^	413.3 ± 18.7 ^ab^	414.5 ± 22.9 ^bc^	1.2 ± 30.5 ^c^	13.9 ± 0.3 ^c^	0.82 ± 0.01 ^a^

Means ± SDs. ^1^ Eutrophic (lean) control rats fed a normocaloric diet (AIN-93M; 3.5 kcal/g); ^2^ overweight/obese controls fed hypercaloric diets (4.1 kcal/g), high in total fat (HF), total sugars (HS) or both (Western; HFS). Control diets ^1,2^ were not supplemented with *Garcinia gummi-gutta* (GGG) rind extract; ^3^ Overweight/obese experimental groups were fed hypercaloric diets described above but supplemented with GGG rind extract (HF+, HS+ and HFS+). Different superscript letters within a column indicate statistical differences (*p* < 0.05).

**Table 3:** Plasma/urine biochemistry from rats fed with experimental diets.

	Glucose	Total Cholesterol	HDL-C	Triacylglycerides	Ketones ^4^
Control ^1^	103.4 ± 8.4 ^b^	126.8 ± 18.7 ^a^	43.0 ± 7.3 ^a^	248.4 ± 65.5 ^a^	3.5 ± 1.8 ^b^
HF ^2^	121.1 ± 16.6 ^a^	116.6 ± 15.8 ^a^	33.6 ± 6.7 ^bc^	144.7 ± 12.2 ^b^	7.1 ± 2.2 ^a^
HS ^2^	118.7 ± 6.1 ^ab^	128.8 ± 12.0 ^a^	35.8 ± 5.0 ^b^	208.1 ± 39.7 ^a^	6.9 ± 4.1 ^a^
HFS ^2^	116.7 ± 20.0 ^ab^	115.2 ± 7.1 ^a^	34.2 ± 6.0 ^bc^	151.5 ± 17.8 ^b^	7.2 ± 3.5 ^a^
HF+ ^3^	103.3 ± 4.9 ^b^	117.3 ± 10.4 ^a^	28.6 ± 3.1 ^c^	142.0 ± 10.6 ^b^	2.1 ± 0.7 ^b^
HS+ ^3^	122.9 ± 18.1 ^a^	121.6 ± 13.1 ^a^	32.9 ± 4.3 ^bc^	146.1 ± 18.9 ^b^	3.5 ± 1.8 ^b^
HFS+ ^3^	108.3 ± 8.1 ^ab^	115.3 ± 5.9 ^a^	27.1 ± 1.8 ^c^	137.0 ± 7.8 ^b^	4.2 ± 1.4 ^ab^

Means (mg/dL) ± SDs. ^1^ Eutrophic (lean) control rats fed a normocaloric diet (AIN-93M; 3.5 kcal/g); ^2^ overweight/obese controls fed with hypercaloric diets (4.1 kcal/g), high in total fat (HF), in total sugars (HS) or both (Western; HFS). Control diets ^1,2^ were not supplemented with *Garcinia gummi-gutta* (GGG) rind extract; ^3^ Overweight/obese experimental groups were fed with the same hypercaloric diets but supplemented with GGG rind extract (HF+, HS+ and HFS+); ^4^ Urine samples. Different superscript letters within a column indicate statistical differences (*p* < 0.05).

**Table 4:** Body fat distribution in rats fed with experimental diets.

	DXA ^4^			Chemical (Soxhlet)		
	Total	Abdominal	Thigh Areas ^5^	Visceral	Hepatic ^6^	Fecal ^7^
Control ^1^	35.1 ± 2.3 ^a^	42.9 ± 2.7 ^abc^	27.5 ± 2.9 ^ab^	68.3 ± 1.6 ^bc^	5.9 ± 0.9 ^c^	1.0 ± 0.4 ^c^
HF ^2^	40.0 ± 4.4 ^a^	51.7 ± 3.8 ^a^	32.9 ± 7.2 ^a^	75.3 ± 1.6 ^a^	15.5 ± 5.7 ^a^	2.0 ± 0.5 ^ab^
HS ^2^	31.8 ± 6.1 ^ab^	42.3± 3.4 ^abc^	26.2 ± 4.3 ^ab^	68.8 ± 3.3 ^bc^	10.5 ± 4.9 ^abc^	1.7 ± 0.3 ^b^
HFS ^2^	37.6 ± 6.1 ^a^	49.3 ± 4.9 ^ab^	31.2 ± 5.1 ^a^	70.9 ± 1.2 ^bc^	12.1 ± 4.6 ^ab^	2.1 ± 0.2 ^ab^
HF+ ^3^	24.9 ± 3.6 ^b^	31.4 ± 10.0 ^d^	20.9 ± 2.2 ^b^	62.9 ± 7.3 ^c^	8.7 ± 2.5 ^bc^	2.4 ± 0.3 ^a^
HS+ ^3^	31.6 ± 7.7 ^ab^	42.3 ± 6.8 ^bc^	26.0 ± 6.5 ^ab^	65.8 ± 7.8 ^bc^	9.3 ± 4.8 ^bc^	2.2 ± 0.5 ^ab^
HFS+ ^3^	31.7 ± 2.7 ^ab^	38.6 ± 0.8 ^cd^	26.0 ±1.2 ^ab^	65.3 ± 6.1 ^bc^	9.1 ± 3.1 ^bc^	2.3 ± 0.5 ^a^

Means (g/100 g BW) ± SDs. ^1^ Eutrophic (lean) control rats fed a normocaloric diet (AIN-93M; 3.5 kcal/g); ^2^ overweight/obese controls fed with hypercaloric diets (4.1 kcal/g), high in total fat (HF), in total sugars (HS) or both (western; HFS). Control diets ^1,2^ were not supplemented with *Garcinia gummi-gutta* (GGG) rind extract; ^3^ Overweight/obese experimental groups were fed with the same hypercaloric diets but supplemented with GGG rind extract (HF+, HS+ and HFS+); ^4^ Dual-energy X-ray absorptiometry (DXA); ^5^ Thigh areas (posterior hip and hind leg). ); ^6^ mean (g/100 g FW) ± SD; ^7^ mean (g/100 g DW) ± SD. Different superscript letters within a column indicate statistical differences (*p* < 0.05).

## Supplementary Materials

The following figures are available online at http://www.mdpi.com/2072-6643/10/5/565/s1, Figure S1. Experimental design (flowchart), Figure S2: Caloric contribution of macronutrients in experimental diets. High fat (HF), high sugar (HS), Western (high fat/sugar; HFS) alone or supplemented with GGG rind extract (HF+, HS+, HFS+).
