# Supplementary material for: Selected Physiological Effects of a Garcinia Gummi-Gutta Extract in Rats Fed with Different Hypercaloric Diets
Source: Nutrients. 2018 May 4;10(5):565. doi: 10.3390/nu10050565 (PMC5986445; doi:10.3390/nu10050565)
Supplement: Supplementary file 1 [file nutrients-10-00565-s001.pdf]

nutrients

MDPI

Selected Physiological Effects of a *Garcinia gummi-gutta* Extract in Rats Fed with Different Hypercaloric Diets

Supplementary Material:

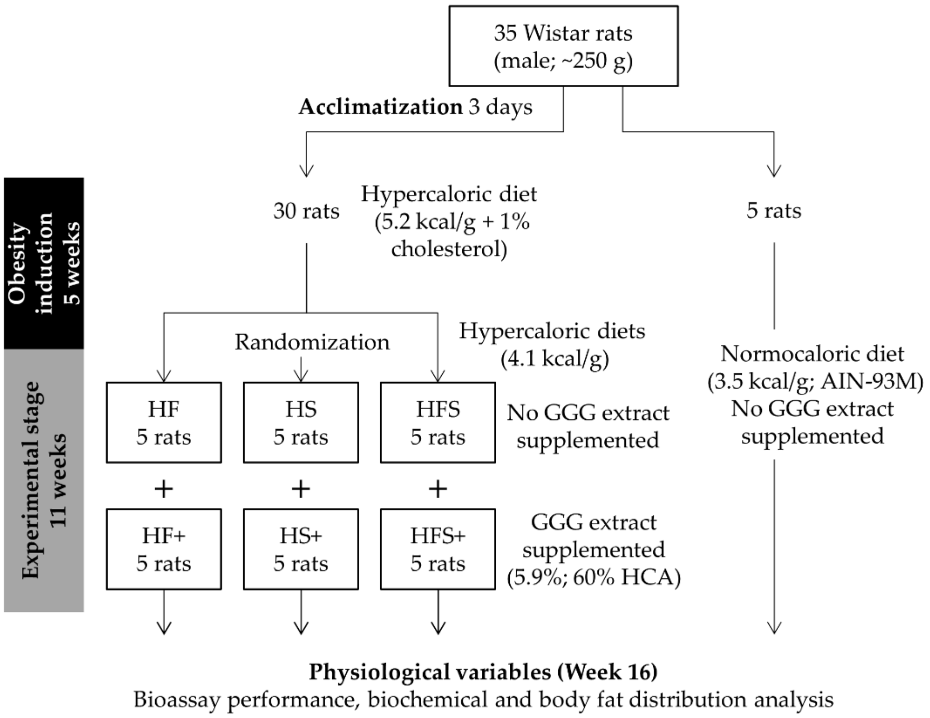

Figure S1. Experimental design (flowchart). *Garcinnia gummi-gutta* rind extract (GGG). Diet nomenclature is described within the text.

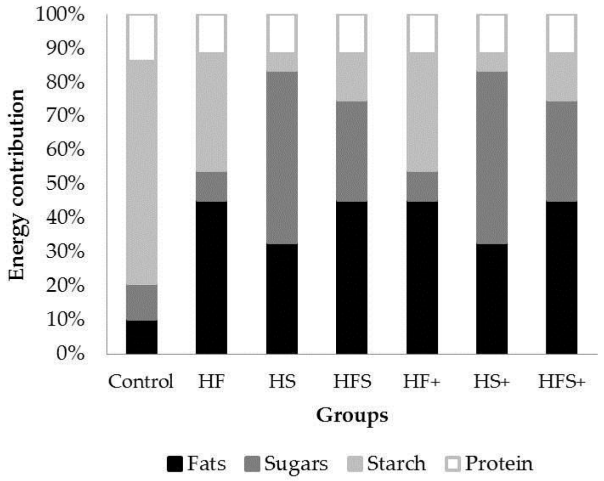

Figure S2. Caloric contribution of macronutrients in experimental diets. High fat (HF), high sugar (HS), western (high fat/sugar; HFS) alone or supplemented with GGG rind extract (HF+, HS+, HFS+).
